# The Checkpoint Program: Collaborative Care to Reduce the Reliance of Frequent Presenters on ED

**DOI:** 10.5334/ijic.5532

**Published:** 2021-06-22

**Authors:** Christine Baird, Yalchin Oytam, Khairunnessa Rahman, Marja Fornasari, Anita Sharma, Jinman Kim, Euijoon Ahn, Rod Hughes

**Affiliations:** 1 Nepean Blue Mountains Local Health District, AU; 2 NSW Ministry of Health, AU; 3 University of Sydney, AU

**Keywords:** integrated care, Emergency Department, frequent presenters, care coordination, case management

## Abstract

**Introduction:**

Growing pressures upon Emergency Departments [ED] call for new ways of working with frequent presenters who, although small in number, place extensive demands on services, to say nothing of the costs and consequences for the patients themselves. EDs are often poorly equipped to address the multi-dimensional nature of patient need and the complex circumstances surrounding repeated presentation. Employing a model of intensive short-term community-based case management, the Checkpoint program sought to improve care coordination for this patient group, thereby reducing their reliance on ED.

**Method:**

This study employed a single group interrupted time series design, evaluating patient engagement with the program and year-on-year individual differences in the number of ED visits pre and post enrolment. Associated savings were also estimated.

**Results:**

Prior to intervention, there were two dominant modes in the ED presentation trends of patients. One group had a steady pattern with ≥7 presentations in each of the last four years. The other group had an increasing trend in presentations, peaking in the 12 months immediately preceding enrolment. Following the intervention, both groups demonstrated two consecutive year-on-year reductions. By the second year, and from an overall peak of 22.5 presentations per patient per annum, there was a 53% reduction in presentations. This yielded approximate savings of $7100 per patient.

**Discussion:**

Efforts to improve care coordination, when combined with proactive case management in the community, can impact positively on ED re-presentation rates, provided they are concerted, sufficiently intensive and embed the principles of integration.

**Conclusion:**

The Checkpoint program demonstrated sufficient promise to warrant further exploration of its sustainability. However, health services have yet to determine the ideal organisational structures and funding arrangements to support such initiatives.

## Introduction

Despite small patient numbers, people who present frequently at Emergency Departments (EDs) consume a disproportionally large share of health care resources [1], particularly where ill-defined conditions, substance-use, homelessness or other behavioural and mental health issues feature in their presentation [2,3,4]. Apart from being costly, reliance on ED for the ongoing provision of care contributes to fragmentation in service provision [5] characterised by poor communication between providers, a lack of service coordination, disruptions to continuity of care [6] and often poor compliance with treatment plans. This is especially problematic for vulnerable patients with multiple needs and chronic conditions that are adversely impacted by psychosocial issues and disadvantage. ED is not equipped to manage many of these patients, partly due to time constraints, resource availability and the organisational context in which ED operates, in addition to the complex and multi-dimensional nature of patient need.

In the area serviced by Nepean Blue Mountains Local Health District (NBMLHD) in New South Wales (NSW) Australia, approximately half of the population has a chronic disease. Over 40% of the population reside in comparatively disadvantaged suburbs where 1 in 8 people delay a medical consultation due to unaffordability. Furthermore 25% of people report difficulty in accessing services (Epidemiological Profile of Local Government Area populations in Nepean Blue Mountains Local Health District). Associated with this, some patients make extensive use of EDs, often as their first port of call, for the provision of ongoing care [7]. To illustrate, those who had made ten or more visits to Nepean Hospital in a year comprised only 0.3% of patients, yet accounted for nearly 3% of presentations (NBMLHD Information Management and Organizational Performance Unit 2017).

While the case for targeting patients who are over-reliant on ED is compelling, efforts to improve the coordination of their care have not always met with success. In NBMLHD, the ‘Care Navigation’ initiative employed nurses specifically to assist patients with chronic and complex conditions to access services in a timely manner and to improve the coordination of care for those presenting to ED. Care Navigation proved no more effective than usual care on a range of measures including hospital presentations. However, the program was somewhat limited in scope. It was entirely hospital-based and did not include case management [7].

There is a growing body of evidence that in order to be effective, in-depth interventions are required [1]. Intervention should reach beyond the traditional boundaries of intra-hospital care to embrace multi-disciplinary case management, providing care which is proactive and which is planned and delivered in the community [8,9,10]. Further, the case has been made for collaborative, whole system care in which navigation of both the medical and social sectors is required. On this view, consideration of mental health problems, housing suitability and the impact of traumatic life events inform the parameters within which presentations of ill-health should be understood and system responses framed. This complements the discourse around ‘social prescribing’ in the primary care setting, as trialled in the UK and which sits alongside medication regimes, allowing the doctor to ‘prescribe’ non-traditional services, for example involvement in community groups to help social isolation [11].

Within its Integrated Care Strategic Framework, NSW Health has recently invested in new models of integrated care, with the stated aim of:

“transforming the health system to routinely deliver person-centred, seamless, efficient and effective care, particularly for people with complex, long term conditions” [12].

This allowed funding for the Checkpoint program to be trialled at Nepean Hospital within NBMLHD. Designed to circumvent the limitations identified in the earlier Care Navigation initiative, Checkpoint sought to improve coordination of care specifically for people who present frequently with the aim of reducing their reliance on ED. This paper describes the program and its outcomes. It complements work reported elsewhere which employed machine learning algorithms to identify patients at risk of re-presentation to ED [13].

## Methods

### Study Design

Ethics approval was obtained from the NBMLHD Human Research Ethics Committee (LNR/16/NEPEAN/110 & LNRSSA/16/NEPEAN/117). We employed a single group interrupted time series design, evaluating year-on-year differences in the number of ED visits for each patient, taking Checkpoint enrolment date as the point of reference and going back 4 years prior to that date. There were a total of 106 patients who had a post enrolment period of at least 6 months, and 51 patients with a period of at least 18 months.

Year-on-year difference score afforded a more direct measure of Checkpoint intervention efficacy because it eliminates the sizable variability in what is a diverse cohort with a broad range of *a priori* ED visit counts. Individual patients were effectively evaluated with respect to their own service utilisation history over the five to six year period before and after intervention. It is worth noting that the difference score has a Gaussian distribution, as opposed to the over-dispersed Poisson distribution of the raw counts. Looking back over a four year period means that any difference observed between the year immediately before and after Checkpoint intervention is considered in the context of previous year-on-year differences, which helps understand whether an observed change can be attributable to the intervention or whether it is within the natural variation of the patient cohort.

### Model of Care

**Figure 1:**
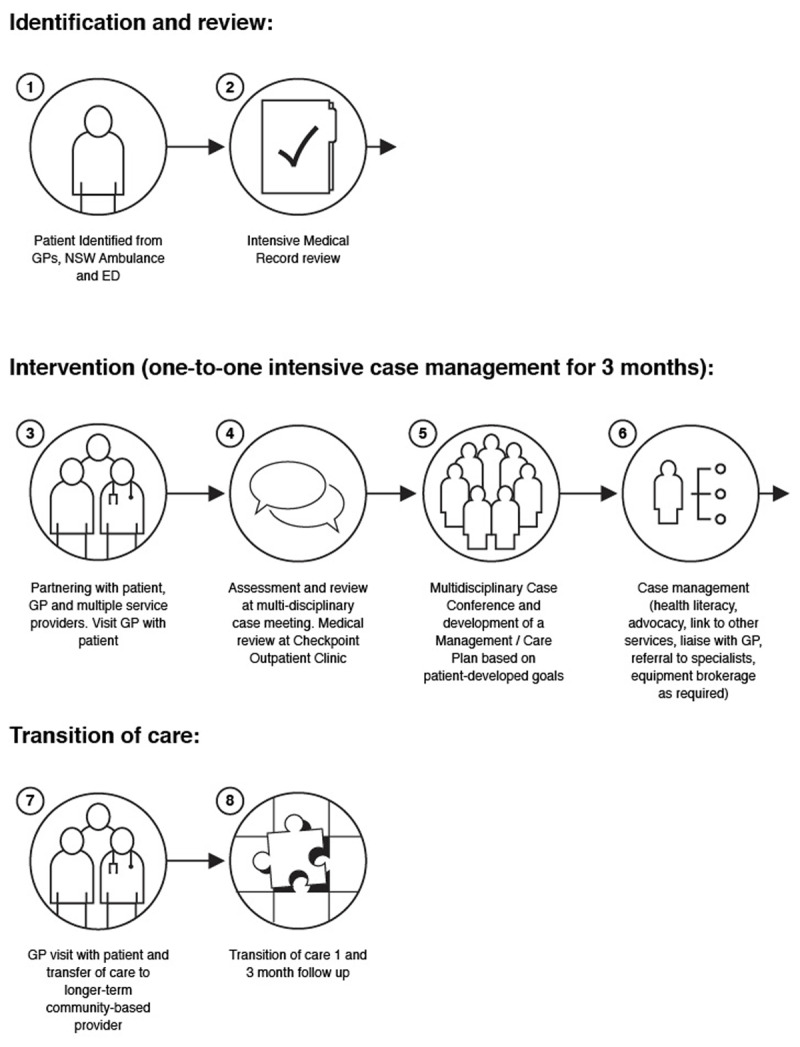
Model of Care.

The Checkpoint intervention comprised three months of targeted, intensive case management irrespective of presenting illness or disease. The Model of Care is depicted in ***Figure 1***. Its core elements were advocacy, client directed goal setting, proactive engagement with client and support services, brokerage of specialist appointments and case conferencing across primary, secondary and tertiary care. These remained constants throughout. However, continuous practice improvements were also made, based on key learnings throughout the two-year implementation period.

While an individual case manager was assigned to each patient, regular clinical reviews and case conferencing promoted care coordination and engagement with GPs. They also enabled multi-disciplinary and multi-service perspectives to be incorporated into care without the need for an additional referral process. Input from the Local Health District’s Aboriginal Health Unit and Multicultural Health Service promoted cultural sensitivity where appropriate. Interpreter services and carer support were utilised as required.

Efforts to promote implementation fidelity included weekly case reviews to monitor client participation and progress as well as team meetings. The latter routinely employed a quality improvement plan-do-check-act cycle to improve program adaptivity in response to problems as they emerged. A steering committee was established, comprising senior managers to promote collaboration between departments. All clinicians met with an independent facilitator to receive group supervision on a monthly basis. Interviews were conducted with a random sample [n = 6] of patient/carers over the course of their enrolment to capture their experiences of patient-centred dimensions of care.

### Enrolment

Patients were eligible for the Checkpoint Program if they had 10 or more ED presentations over the course of a year. This number was chosen somewhat arbitrarily, but it was in the mid-range of definitions found in the literature [14]. Other requirements were that they be under 70 years of age, had ongoing health issues, were attending multiple services for care and had complex needs. Patients were excluded if receiving post-operative or antenatal care, suffering acute conditions that were likely to need time limited interventions, had a single diagnosis or were receiving palliative care in the last 12 months of life, renal dialysis or chemotherapy. The age limit was adopted as several programs were already in place for older patients.

Patients meeting the eligibility criteria were referred directly by the ED Department and invited by telephone to enrol. Written consent was obtained at their initial appointment. 289 patients met inclusion criteria. However, fifty could not be contacted and 49 were well managed by other services. 32 people declined; a further 26 moved out of area while ten were too unwell to participate. Seven withdrew, usually soon after initial enrolment. Insufficient post-intervention data was available for further seven participants, as they had only recently completed the program. Two patients died after initial contact. This resulted in a final N = 106.

### Data Analysis

We collected data on participant characteristics and the number of individual ED presentations on a monthly and yearly basis. Program engagement was operationalized in terms of the proportion of cases for which case conferences were held and GP involvement achieved, as well the number of patient-identified goals set and the proportion achieved. Presentation savings were derived from the value currently placed by NSW Health on individual ED presentations. The patient experience was captured from interviews with a small randomly selected sample of patients [n = 6] while they were still enrolled. Patient experience questionnaires were also completed by 24 participants immediately post program.

Repeated measures ANOVA and t-tests were used to compare year-on-year differences in ED presentations before and after intervention. We also collected data on the costs attributable to these ED presentations. All analyses were performed using R Statistical Software. Patient information for Checkpoint was linked to NSW Emergency Department (EDDC), Hospital Admissions (APDC), and Registry of Births and Deaths datasets. Local Health Districts responsible for the provision of Integrated Care programs funded by the NSW Ministry of Health periodically provided Integrated Care cohort information to the Centre for Health Record Linkage (CHeReL). De-identified linked data was then provided to the System Integration Monitoring and Evaluation team within the System Information and Analytics branch at the NSW Ministry of Health for evaluation. The data linkage upon which this evaluation is based spans a period of eight-and-a-half years, from 1st January 2010 to 30th June 2018 and captures ED presentations, per month and year, anywhere in NSW. All patient data was de-identified prior to analysis.

## Results

### Participants

The Checkpoint Program targeted patients under 70 years of age who had multiple (10 plus) unplanned re-presentations to the Nepean hospital’s ED in 12 months. This resulted in 106 participants, of whom 55% were male with a median age of 42 years. 25% of participants were in their 20s and a further 25% were in their 50s. 82% [n = 87] were born in Australia. 15% [n = 16] were of Aboriginal ancestry. The majority had either never married [n = 57; 53.8%] or were separated/divorced [n = 19; 18%]. Only a minority were married or in a de facto relationship [n = 28; 26.4%]. Two participants had been widowed [n = 2; 1.8%]. The most frequently occurring diagnoses at ED presentation are listed in ***Table 1***.

**Table 1:** Top twenty diagnoses upon presentation at ED [ICD10V6].


	N	%

Other and unspecified abdominal pain	218	10.1%

Chest pain, unspecified	105	4.8%

Other drugs, medicaments and biological substances	67	3.1%

Suicidal ideation	57	2.6%

Mental disorder, not otherwise specified	57	2.6%

Unspecified personality and behaviour disorder	54	2.5%

Chronic obstructive pulmonary disease, unspecified	53	2.4%

Unknown and unspecified causes of morbidity	50	2.3%

Dyspnoea	50	2.3%

Asthma, unspecified	37	1.7%

Malaise and fatigue	36	1.7%

Nausea and vomiting	35	1.6%

Elevated blood glucose level	30	1.4%

Hypoglycaemia, unspecified	30	1.4%

Procedure not carried out, unspecified reason	29	1.3%

Unspecified dorsalgia, site unspecified	27	1.2%

Mental and behavioural disorders due to alcohol	26	1.2%

Other and unspecified convulsions	23	1.1%

Anxiety disorder, unspecified	22	1.0%

Headache	22	1.0%


**Figure 2:**
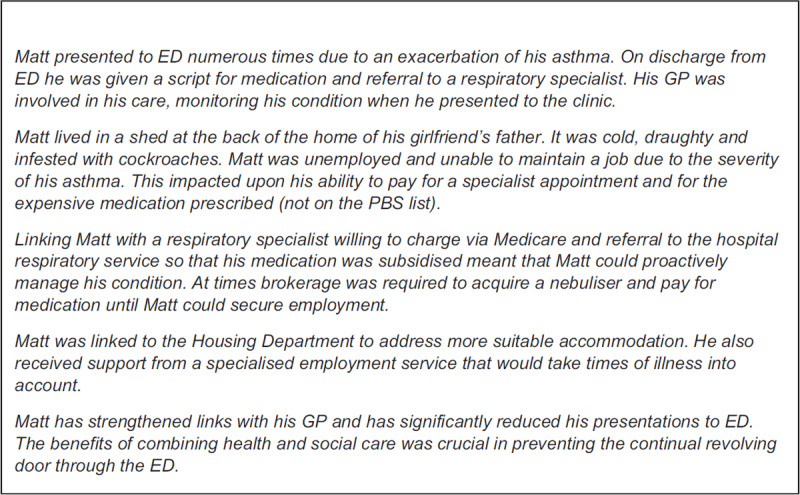
The Patient Journey.

Initial assessment occurred immediately after enrolment, whereupon mental health was the most commonly identified patient need (***Table 2***). Two-thirds of participants [n = 71] identified four or more needs. A high percentage of Checkpoint patients had experienced past trauma, and had a functional disability exacerbated by their social disadvantage. 85% were receiving a government welfare benefit with 53% of these patients experiencing financial difficulty. This contributed to problems with medication compliance and keeping appointments [***Figure 2***: The Patient Journey].

**Table 2:** Most frequently identified patient needs at assessment.


	N	%

Mental health	43	40.6

Pain management	26	24.5

Accommodation [Inc. homelessness]	24	22.6

D&A issues	22	20.8

Financial	22	20.8

Social isolation	21	19.8

Diabetes	15	14.2

Dietitian review	14	13.2

GP	12	11.3

Women’s health	12	11.3


Approximately 70% of the patients attended the ED by ambulance. Notably this patient group were also responsible for a high number of call outs to the Ambulance Service of NSW that did not result in transportation to the ED. Although 77% of the patient cohort had a listed GP, a substantial proportion had little to no contact with their GP within the last 12 months immediately prior to enrolment.

### Program Engagement

As a result of the Checkpoint program, multidisciplinary case conferences, including the patient (with carer when relevant), were held in 89% of cases [n = 91]. 79% of participants [n = 83] had active involvement from their GPs. The number of patient-identified goals set in management plans ranged from 1 to 21 with a median of 7. The median percentage of goals achieved was 90%.

### Presentation Rates

**Figure 3:**
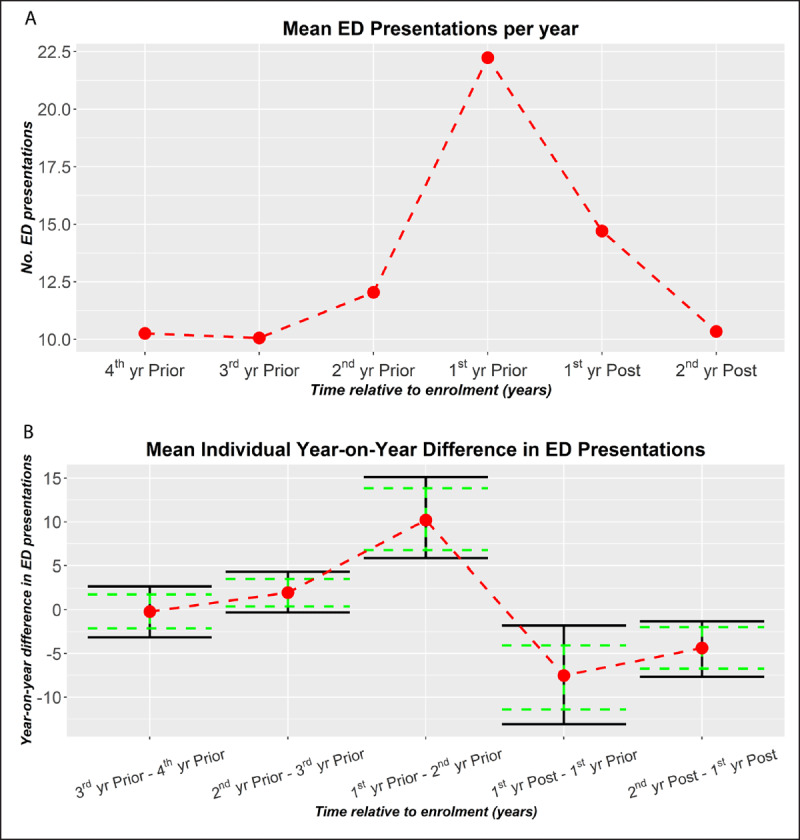
Trend in ED Presentations of the Checkpoint Cohort. **A**. The average ED presentations of the Checkpoint cohort with a post enrolment period of at least 18 months [N = 51]. For patients with 18-24 month post enrolment period, pro rata adjustments are made to the 2^nd^ yr Post ED presentation counts. **B**. The average year-on-year difference in ED presentations for the same Checkpoint Cohort (A). Solid (black) bars denote 95% CI. Dashed (green) bars are adjusted to indicate statistical difference (p-value .05) for paired comparisons [18].

As depicted in ***Figure 3***, results show two consecutive year-on-year reductions following intervention (ANOVA, F-Value = 9.605, Pr(>F) = 4 × 10^–7^; t-tests, effect size = –7.5, t = –2.70, p-value = .010 and effect size = –4.4, t = –2.60, p-value = .012, respectively). In contrast, prior to intervention there appears to be an increasing trend in presentations, the bulk of which occurs in the 12 months leading to enrolment. By the second year, there is a net reduction of 11.9 presentations per patient per annum. From a peak of 22.5 presentations per patient, this amounts to a relative drop of 53%.

**Figure 4:**
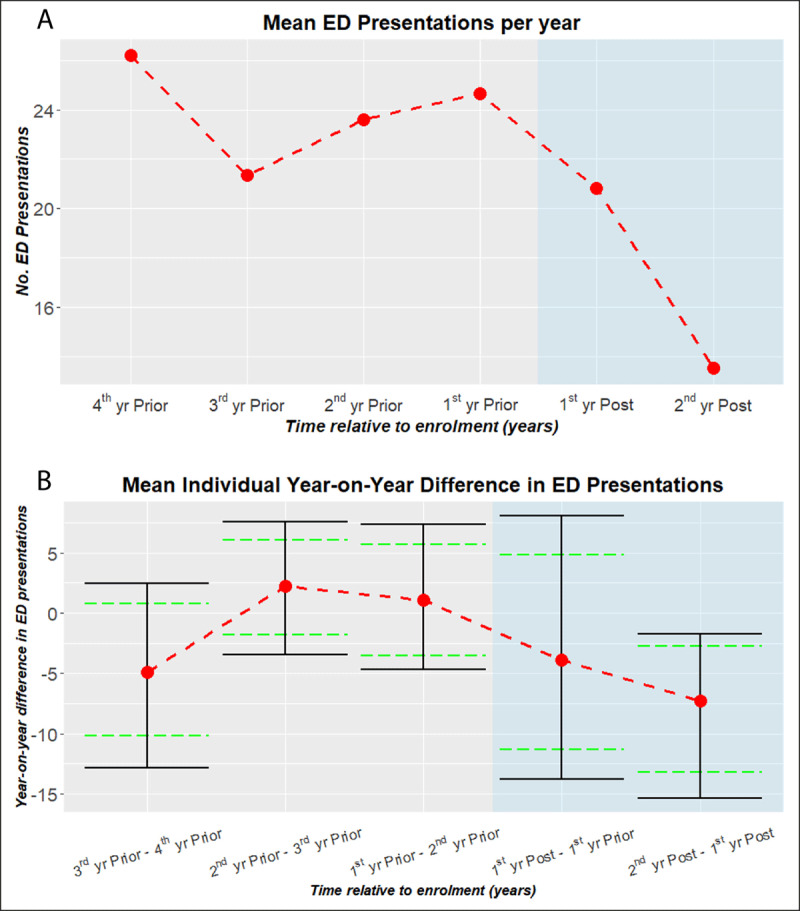
Trend in ED Presentations of the Checkpoint Cohort for persistent frequent presenters. **A**. The average ED presentations of the Checkpoint cohort with a post enrolment period of at least 18 months [N = 15]. Persistent frequent presenters are defined as having ≥ 7 ED presentations each year for the last four years. For patients with 18–24 month post enrolment period, pro rata adjustments are made to the 2^nd^ yr Post ED presentation counts. **B**. The average year-on-year difference in ED presentations for the same persistent frequent presenters cohort (A). Solid (black) bars denote 95% CI. Dashed (green) bars are adjusted to indicate statistical difference (p-value .05) for paired comparisons [18].

**Figure 5:**
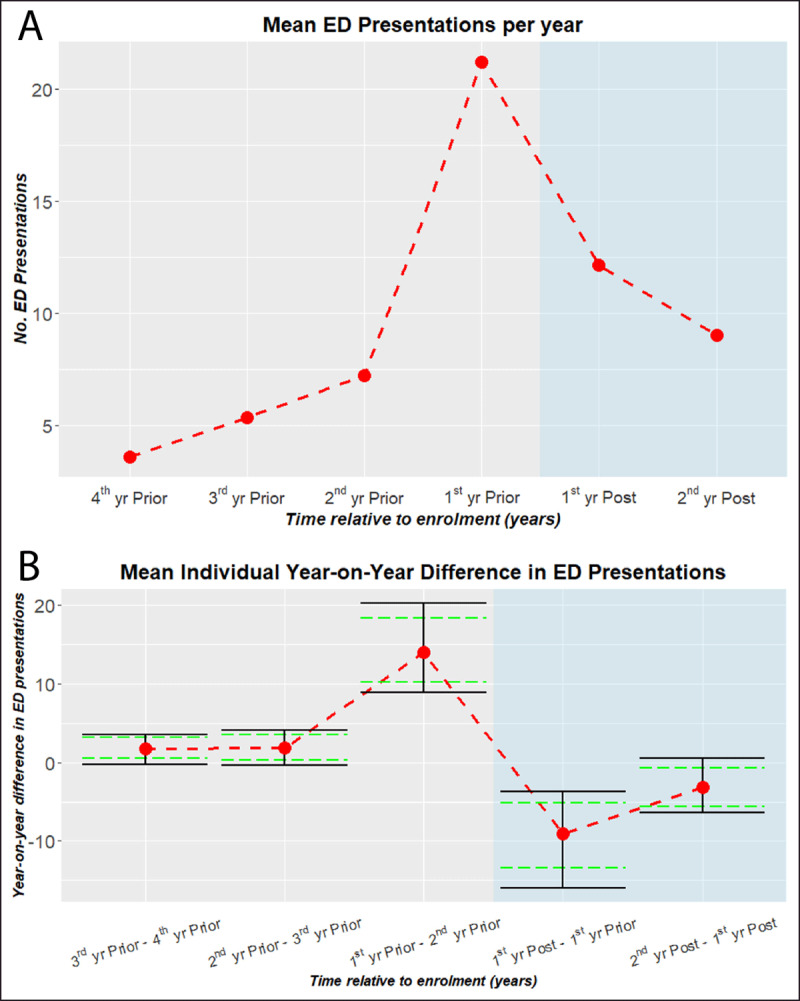
Trend in ED Presentations of the Checkpoint Cohort for non-persistent frequent presenters. **A**. The average ED presentations of the Checkpoint cohort with a post enrolment period of at least 18 months [N = 36]. Non-persistent frequent presenters are defined as not having ≥7 ED presentations each year for the last four years. For patients with 18-24 month post enrolment period, pro rata adjustments are made to the 2^nd^ yr Post ED presentation counts. **B**. The average year-on-year difference in ED presentations for the same non-persistent frequent presenters cohort (A). Solid (black) bars denote 95% CI. Dashed (green) bars are adjusted to indicate statistical difference (p-value .05) for paired comparisons [18].

Within this cohort of patients who had a post enrolment period of at least 18 months, there were two dominant modes in the ED presentation trends (***Figures 4*** and ***5***).

One group (n = 15) had a steady pattern with ≥7 presentations each of the last four years. The other group (n = 36) had an increasing trend in presentations, peaking in the 12 months immediately preceding enrolment. Following the intervention, both groups demonstrated significant reductions (***Figures 4*** and ***5***). In relative terms, the reduction at 18–24 months post enrolment was more pronounced in the persistently frequent ED presenter group.

### Presentation Savings

Australian governments have a costing agreement, which has established a common unit to measure health service activity (National Weighted Activity Unit, or NWAU). It forms the basis for comparing and valuing each public hospital service (whether it is an admission, emergency department presentation or outpatient episode), by weighting it for its clinical complexity [15]. NSW Health values the average ED presentation at 0.127 NWAU, and the current dollar value of NWAU is set at $4713. A reduction of 11.9 presentations amounts to $7122, per patient for the twelve-month period.

### The Patient Experience

Interviews were conducted with a small randomly selected sample of patient/carers [five patients and one carer] while patients were still enrolled in Checkpoint. 24 participants also completed questionnaires [patient-reported experience measures or PREMs] post program. The predominant reason as to why clients came into ED had pertained to feelings of helplessness and inability to cope with their medical conditions and/or social situations at hand. Clients described a recurrent cycle of stressors from both poorly controlled medical conditions and negative life circumstances, which escalated their need to attend the ED repeatedly.

At interview, all clients reported significantly reduced visits to ED since Checkpoint enrolment, citing better pain management and/or control of their medical issues as well as significant improvements in social connections and supports within the community. Clients mentioned that working on small goals with the various supports that had been pulled together by Checkpoint made improving health and social outcomes achievable. Two clients also noted the fast-tracking of their care in the community whereby previously difficult issues were progressed as a result of the case managers’ proactive lobbying. Questionnaire feedback was also very positive with average ratings in the order of 90% on dimensions such as ease of making appointments, involvement of family/friends, access to services and ease of understanding information.

## Discussion

Reductions in ED presentation rates are desirable for any service seeking to optimise the utilisation of scarce health care resources, and certainly in NBMLHD where the demand for services continues to grow and the ED struggles to meet the benchmarks for emergency treatment performance. This is despite the significant measures already implemented such as increasing physical space, the deployment of additional ED staff and attempts at improving in-hospital care navigation.

It must be kept in mind that this was a single group interrupted time series design with a relatively small sample size, constrained by the project’s funding stipulations and the exigencies of service provision. The absence of a control group precluded any definitive attribution of results to the intervention, since other explanations for the decline in presentation rates could not be ruled out. For example, it could be that over the course of the project, participants grew tired of growing waiting times at ED, or that they were able to access other providers of health care services, or they simply improved in the management of their conditions independently of the intervention. These explanations may be unlikely, given the illness trajectories of this patient group and the trend in presentation rates observed over an extended period of time.

Another challenge to our interpretation that the intervention is the cause of the reduction in ED presentations is regression to the mean. It could be that some patients had a relatively acute period in the lead up to enrolment and naturally regressed to their base level since. Again, given that patients with a stable and high frequency of presentations over a four-year period (i.e. persistent frequent presenters) showed the largest reduction post enrolment, regression to the mean is an unlikely explanation for the entirety of the effect that we observe. It is also known that persistent frequent presenters may receive more benefit from case management, and increased continuity of primary care than transient frequent presenters [16], which fits the intervention we provided.

Two years’ experience with the implementation of Checkpoint suggests some of the more important ingredients for success. Elements of intensive case management were combined with best practice in care coordination [17] in which staff took explicit and active responsibility for that role. They built relationships with other providers, clarifying expectations and the contributions each would make. They also established agreed ways of sharing information and took special care at transition points, making sure that patients could negotiate care pathways and associated appointment processes, especially when these crossed organisational boundaries. A multidisciplinary approach to care proved valuable, bringing expertise and knowledge together for a comprehensive and collaborative approach, directing care at goals the patient had developed. These were often not medical in nature e.g. improving education, accommodation, reconnecting with cultural roots, developing spiritually, and improving social relationships. This dictated that assistance be provided holistically and organized around a broader understanding of patients’ need, transcending the silos in which health services are traditionally organised.

One of the key challenges for the Checkpoint program entailed being able to provide intensive support without promoting participant dependency. Partnerships with other providers proved important here. Case managers were careful not to change existing routines, trying to work within the framework provided by patients themselves – arranging to see them while they were attending other clinics for example. This meant that when the intervention was withdrawn, existing structures were still in place. Case managers also took time to coach participants in communicating their needs and in negotiating with health care providers on their own behalf. When this occurred, clients reported a sense of independence and improved self-confidence in being able to express their needs.

People who present frequently at ED are commonly labelled as difficult or non-compliant [1]. Checkpoint challenged this perspective by firstly seeking to understand their complex life circumstances, asking *why* they are presenting frequently and what barriers these patients face. The proactive engagement and involvement of the patient and their carer/s was an essential component of care planning for each participant. Navigating an increasingly complex health system is difficult enough, but more so when compounded by factors such as multiple specialist appointments, multiple medications, poor health literacy, financial burden, unstable accommodation, mental illness and dependence on alcohol and other drugs. In practical terms this meant a pro-active intervention style: being prepared for example to accompany patients to appointments, to negotiate on a patient’s behalf about specialist fees and having the flexibility to work outside of usual service parameters. It also called for creative ways of maintaining contact when chaotic lifestyles made normal modes of communication difficult. Access to a level of brokerage to assist patients with the purchase of equipment and medication also proved valuable.

Approximately 80% of participants benefited from the active involvement of their GP in the program. This was a significant achievement given that prior to intervention, many reported little or no contact with a medical practitioner; others had been ‘doctor shopping’ between practices. Accompanying patients to GP appointments proved a useful strategy to ensure patients connected with an appropriate primary medical care provider. Once this relationship was established, the GP became a pivotal player for the provision of ongoing medical management in the community, the most appropriate setting for ongoing client care, as well as providing input to multi-disciplinary case discussions.

Initiatives in co-ordinated care can be costly [7]. Considering establishment costs, Checkpoint was no exception. It is widely accepted that innovation and efficiencies do not necessarily go hand in hand, especially in the short term. They require time to be explored, thus increasing initial outlays. It is reasonable to expect that total savings would be greater once the model was embedded and clinician caseloads as well as organisational systems and processes were optimised. When reductions in the costs of presentations and admissions [approx. $1.5 m over 2 years] are compared with ongoing program expenditure we observe a neutral return on investment, noting set up costs were additional to this expenditure. However, this takes no account of indirect benefits to patients such as improvements in their health and well-being which might accrue over the longer term as a result of improvements in their circumstances and increased capacity for self-management. Nor did we quantify any shifting of costs to other providers, or indeed any positive externalities, as this too was beyond the scope of the project. These no doubt included the benefits associated with removing duplication in consultations and tests and reduced demand for ambulance services.

With the recent advance in data analytics, there are many opportunities to better understand the characteristics of the frequent presenters of ED. These analytical tools have powerful population level capabilities such as data mining and predictive modelling (e.g. prediction of at-risk patients [13]) to support evidence-based decisions about the optimal allocation of resources for interventions such as Checkpoint. This should assist in reducing the overall cost of the program and providing opportunity for scalability across settings.

Questions remain about the ideal duration and intensity of intervention. Checkpoint was delivered over a 3 month period with provision for one and three months follow-up. By comparison, a similar program, ‘Bridges to Care’ was delivered over a period of just 60 days with similar program inputs [19]. Other questions concern which elements are the most important contributors to success, whether outcomes are improved by the provision of follow-up support sessions and whether benefits are sustained over a longer period. These suggest avenues which further research could usefully explore.

## Limitations

Sample size was small and the follow-up period relatively short. Also, in the absence of a comparison group, results should be interpreted with caution. A similar caveat applies to the financial analysis undertaken, limited as it was to the costs directly incurred by the program compared to the savings imputed from the observed reductions in presentations.

## Conclusion

This project lends some weight to a growing body of evidence that efforts to improve care coordination, when combined with proactive case management in the community, can impact positively on ED re-presentation rates, provided they are concerted, sufficiently intensive and embed the principles of integration. These kinds of interventions are of course not new, yet successful program implementation is far from guaranteed [20,21] and faces a number of ongoing challenges. While the need for overarching governance has been canvassed, health services have yet to determine the ideal organisational structures, funding arrangements and performance measures to support these initiatives [10,22,23], and in this connection how best to circumvent the traditionally compartmentalised way in which health services operate; also, how to assign value to indirect patient care and program benefits, not just to patients but those that accrue to other service providers.

Since the completion of the Checkpoint pilot, these findings have informed the model subsequently adopted in other parts of NSW. Future iterations and adaptations of this program will provide opportunities for studies with larger sample sizes and suitably matched comparison groups. More thoroughgoing economic evaluations are also required.
